# The Public Health Responsibility Deal: Using a Systems-Level Analysis to Understand the Lack of Impact on Alcohol, Food, Physical Activity, and Workplace Health Sub-Systems

**DOI:** 10.3390/ijerph15122895

**Published:** 2018-12-17

**Authors:** Cécile Knai, Mark Petticrew, Nick Douglas, Mary Alison Durand, Elizabeth Eastmure, Ellen Nolte, Nicholas Mays

**Affiliations:** Faculty of Public Health and Policy, London School of Hygiene & Tropical Medicine, 15–17 Tavistock Place, London WC1H 9SH, UK; mark.petticrew@lshtm.ac.uk (M.P.); nick.douglas@lshtm.ac.uk (N.D.); mary-alison.durand@lshtm.ac.uk (M.A.D.); elizabeth.eastmure@lshtm.ac.uk (E.E.); ellen.nolte@lshtm.ac.uk (E.N.); nicholas.mays@lshtm.ac.uk (N.M.)

**Keywords:** systems approach, systems thinking, public-private partnership, alcohol, food, workplace health, physical activity

## Abstract

The extent to which government should partner with business interests such as the alcohol, food, and other industries in order to improve public health is a subject of ongoing debate. A common approach involves developing voluntary agreements with industry or allowing them to self-regulate. In England, the most recent example of this was the Public Health Responsibility Deal (RD), a public–private partnership launched in 2011 under the then Conservative-led coalition government. The RD was organised around a series of voluntary agreements that aim to bring together government, academic experts, and commercial, public sector and voluntary organisations to commit to pledges to undertake actions of public health benefit. This paper brings together the main findings and implications of the evaluation of the RD using a systems approach. We analysed the functioning of the RD exploring the causal pathways involved and how they helped or hindered the RD; the structures and processes; feedback loops and how they might have constrained or potentiated the effects of the RD; and how resilient the wider systems were to change (i.e., the alcohol, food, and other systems interacted with). Both the production and uptake of pledges by RD partners were largely driven by the interests of partners themselves, enabling these wider systems to resist change. This analysis demonstrates how and why the RD did not meet its objectives. The findings have lessons for the development of effective alcohol, food and other policies, for defining the role of unhealthy commodity industries, and for understanding the limits of industry self-regulation as a public health measure.

## 1. Introduction

The extent to which government should partner with business interests such as the alcohol, food, and other industries in order to improve public health is a subject of ongoing debate [1,2,3]. Governments vary in the extent to which they intervene in the market in pursuit of public health goals, though a common approach involves developing voluntary agreements with industry or allowing them to self-regulate, as opposed to introducing legislation. In England, the most recent example of this as a policy measure has been the Public Health Responsibility Deal (RD), a public–private partnership launched in 2011 under the then Conservative-led coalition government. An independent evaluation of this initiative was undertaken, the separate components of which have been published elsewhere [4,5,6,7,8,9,10,11,12,13].

This paper brings together for the first time the main findings and implications of the evaluation using a systems approach. We are providing this analysis because we want to understand better why each aspect of the RD that we have studied seems to have had little or no positive impact on public health. Bringing together the findings of the evaluation components should allow us to present a system-level explanation of the RD, and of its lack of public health impact. It should be noted that this analysis takes a public health perspective since the RD was introduced from the outset as a public health policy innovation; a business perspective on the RD and its success may reach different conclusions. It is also timely to do this given that partnerships between industry and public health bodies are ongoing and controversial, as evidenced by the partnership announced in September 2018 between the alcohol industry body Drinkaware and the public health agency Public Health England.

A systems approach can provide new insights into such relationships. In public health, such an approach aims to provide a comprehensive overview of the drivers of complex issues such as excess alcohol consumption, poor diet and physical inactivity, and sees them as embedded within, and shaped by, wider political, institutional, and cultural factors [14,15,16,17,18]. According to the seminal work by the environmentalist and systems theorist Donella Meadows, a system can be conceptualised as a set of elements (such as data, actors, institutions) interconnected (through information flows or feedback loops) in such a way that it produces its own pattern of behaviour over time, and with a specific function or purpose (characterised by the system’s goals, or paradigm—such as to produce health, or to achieve business objectives) [19,20]. A “high-functioning” system is said to be characterized by [19]: (1) resilience, the ability to persist within a variable environment; (2) adaptivity, the capacity to diversify and evolve in response to external pressures; and (3) hierarchy, the ability to arrange subsystems to support the functioning of the system, and build its resilience [19]. Note that in this context the description “high-functioning” does not have either positive or negative connotations for public health.

**Figure 1:**
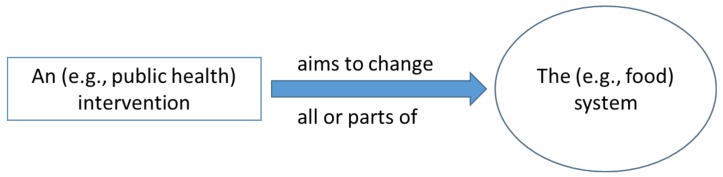
How a public health intervention is conventionally conceptualised.

Source: authors

These properties should be considered when thinking of a public health intervention which aims to change the behaviour of an individual (or a group of individuals) in the system. Yet a major challenge posed by this definition is that we often imagine and conceptualise an intervention as being *external* to the target population. For example, an intervention can consist of testing a hypothesis by imposing a new activity (e.g., extra physical education at school), providing a piece of information (e.g., leaflets), or a new physical environment (e.g., how a supermarket is configured) to see whether it has an effect, or by observing behaviours. In all cases, if one were to draw a picture of the intervention, it would most usually be conceptualised as a new action imposed from the outside, as illustrated in Figure 1.

**Figure 2:**
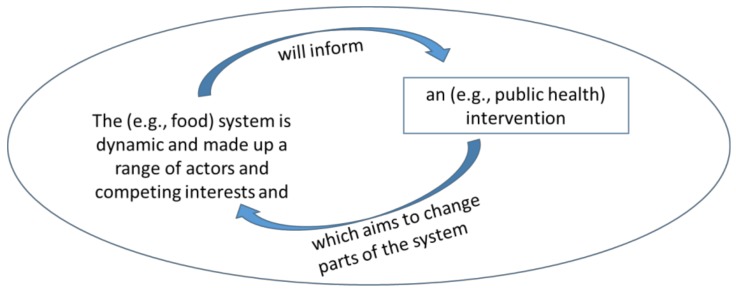
A systems perspective on interventions, where the intervention is also part of the system.

Source: authors

Yet, an intervention is, of course, also designed by actors within a system (e.g., policy advisers, academics, non-governmental organisations (NGOs), business, or others), who have a stake in the success of this intervention, defined in terms of meeting public health aims, increasing professional prestige, relieving funding pressures, etc., and also therefore in how it is designed (e.g., evidence-based, measurable, reproducible). They will also, crucially, define the components of the intervention. Systems thinking helps to visualise the intervention as being part of the system (Figure 2) and encourages the evaluator to ask questions with respect to who designed the intervention, how was it designed and why, and how the intervention was implemented. 

The RD was organised around a series of voluntary agreements that aimed to bring together government, academic experts, and commercial, public sector and voluntary organisations to commit to pledges to undertake actions potentially of public health benefit. The main pledges covered alcohol, food, physical activity and health at work. These corresponded to the four “networks” of the RD [21]. Upon committing to a pledge, partner organisations were asked to submit a delivery plan, outlining the specific actions they would take to implement this pledge. In addition, partners also committed to monitoring against agreed indicators, and to providing annual progress reports. At time of writing (May 2017), 776 organisations were reported to be partners in the RD. 

Thus the “system” relevant to the RD comprises the alcohol, food, physical activity and workplace networks, and specifically the full range of activities from production, processing, and marketing, to consumption and disposal of goods, and the people and institutions that generate or constrain change in the system. A systems approach theorises that the effect of a given input or intervention depends on other conditions in the system, rather than on the mechanism of a particular intervention—or group of interventions—alone [22]. Thus, in this paper we bring together the findings of individual parts of the evaluation, using a systems perspective. The alternative would be to consider the evaluation as consisting of a set of linked but separate components, assessing different interventions within the RD (such as different pledges). However, the RD itself aimed at something larger: to change the alcohol, food, health at work, and physical activity sub-systems by encouraging action from within, such as by businesses. According to the former Secretary of State for Health, Andrew Lansley, under whom the RD was established, the aim of the RD was that “*by working in partnership, public health, commercial and voluntary organisations can agree practical actions to secure more progress, more quickly, with less cost than legislation*” [21].

## 2. Methods and Materials

The RD evaluation (2012–2015) involved several strands of research [23]. The data from these individual strands are drawn together in the current paper to help explain the mechanisms and effect of the RD as a whole, through a systems lens.

The main individual strands were: the development of an initial logic model of the RD, based on literature review, and individual interviews with selected stakeholders; a scoping review of the literature on public–private partnerships (PPPs); a qualitative study to explore informants’ views and experiences of the RD’s development, implementation and achievements; evidence syntheses of each pledge, by network (e.g., alcohol, food); analyses of progress on achieving pledges overall and analyses of specific pledges; case studies of how individual organisations implemented their pledges; international comparative case studies of similar voluntary agreements; and an analysis of how the RD was presented in the mass media [4,8,10,11,12,13,23]. Table 1 shows how these components contributed data to the systems-level analysis in the current paper.

We present the findings of the RD evaluation using four questions [19,28]. These questions can help explore the functioning of a complex intervention or complex system. We use this framework to explore how the RD interacted with the alcohol, food, physical activity and workplace health systems, and to help understand its lack of effect, through a systems lens: The questions as applied to the RD are:

(1) What are the causal pathways involved in the RD and how did they help or hinder it?
(2) What were the RD structures, processes and interests at play?
(3) What were the feedback loops, and did they suppress or potentiate effects of the RD on the outcomes of interest?
(4) How resilient was the system to change and what was its ability to “absorb” externally directed change?

We address these questions in turn, drawing on the findings from the RD evaluation as relevant.

Finally, we employ qualitative systems dynamic modelling (SDM) to illustrate the RD as a system [15]. Although SDM is usually a quantitative method, it has been successfully employed, for example, to support housing policy stakeholders to map the links between environmental, economic, social and health outcomes and to improve their understanding of housing as a complex system [15] or to analyse an urban water management system [14].

Causal loop diagrams (CLDs) can help to visualise a system [27] and clarify the individual components of the RD, and any interconnections between them [29].

## 3. Results

### 3.1. What Were the Causal Pathways Involved, and How Did They Help or Hinder the Intervention (the Responsibility Deal)?

The RD was highly complex in that it was made up of many interacting components, each at different stages of development, operating at different levels, with different potential outcomes and mechanisms, and implemented in very different contexts (i.e., across the alcohol, food, physical activity, and health at work networks) [23]. The RD could be seen either as a single intervention (that is, the overall RD policy), or around forty individual pledges guiding a set of specific interventions. A simple outcome evaluation to assess whether the RD had “worked” would have been potentially misleading and probably impossible, with at least three major challenges—the lack of a suitable comparator or counterfactual; the lack of a “pre-RD” baseline against which to analyse impact; and the low likelihood of proximal changes in population health relevant to the RD.

**Figure 3:**
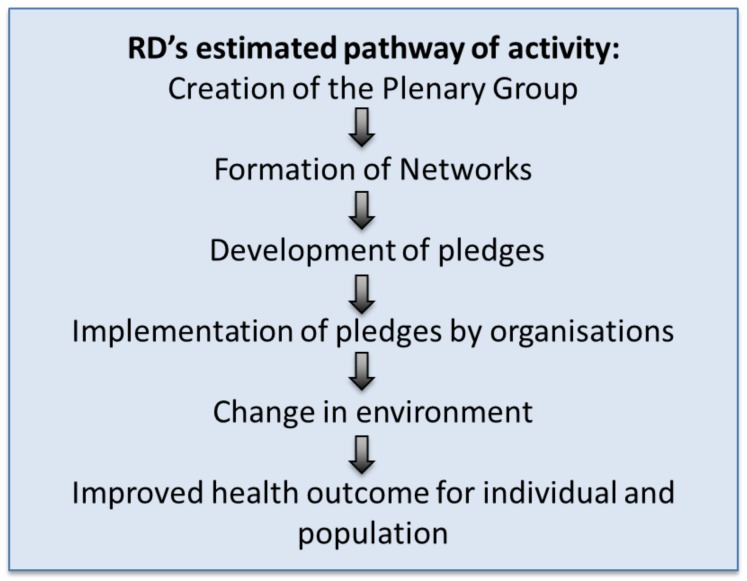
Simple logic model. RD: the Public Health Responsibility Deal.

Source: adapted from Petticrew et al. 2013 [23]

With these evaluation challenges in mind, we developed a logic model to clarify the underlying assumptions about how the RD might work to improve population health since it was presented as a public health policy (simple version in Figure 3) [23]. Most importantly, not only did the logic model show the assumed causal pathway between any action taken as part of the RD, and its intended outcomes, but also it implied that if certain steps were not taken, or were poorly implemented, then there was little or no likelihood that the action would change outcomes further along the causal pathway.

The logic model identified the practical starting point of the RD to be the formation of an RD plenary group of senior representatives from the business community, NGOs, and public health organisations to oversee the RD [21]. Networks were then formed, followed by the development of initial pledges. The subsequent negotiation, agreement and implementation of pledges were important stages along the pathway by which the RD was assumed to affect health. The implementation of the pledges were meant to result in changes in the environment, individual behaviours, and in turn, to improved health. Finally, it was assumed that the cumulative effect of the individual responses would lead to population level health improvement [23]. Indeed the official RD launch document illustrated these assumptions as it was explicitly about individual behaviour change and individual health, as outlined in the core commitments of the RD (Box 1) and stated that “*The Responsibility Deal taps into the potential for businesses and other organisations to improve public health and help to tackle health inequalities through their influence over food, physical activity, alcohol, and health in the workplace*” [21]. Despite the apparent commitment to population level health gain, there was a large reliance on individual behaviour change which was most unlikely to lead to game-changing system effects.

Box 1 **The core commitments of the Public Health Responsibility Deal. Source: The Public Health Responsibility Deal launch document 2011 [21].**

The business community, voluntary sector and NGOs have already done a great deal to help people achieve a healthier diet, increase their levels of physical activity, drink sensibly and understand the health risks of their lifestyle choices. Signatories to the Public Health Responsibility Deal will work in support of the following core commitments in relation to their customers and staff, where relevant.

1. We recognise that we have a vital role to play in improving people’s health.

2. We will encourage and enable people to adopt a healthier diet.

3. We will foster a culture of responsible drinking, which will help people to drink within guidelines.

4. We will encourage and assist people to become more physically active.

5. We will actively support our workforce to lead healthier lives.

The logic model helped identify several key questions regarding the intervention. The first question raised was whether the chosen pledges and the actions associated with implementing them were ever likely to affect population behaviour and thence population health in a positive way. Crucially, it also raised the question of whether the four networks were the “right” networks to affect public health, and whether the individuals leading the networks were appropriate to the task. Finally, it raised the question of whether the RD would likely lead to “more progress, more quickly, with less cost than legislation” [21] and other policy changes. The following sections help address these questions. 

### 3.2. What Were the Responsibility Deal Structures, Processes and Interests at Play?

#### 3.2.1. Structures Driving the RD

As noted above, the RD plenary group was an important initial structure as it defined the nature of the networks and in turn, the pledges. At last update (24 September 2013), the Plenary Group comprised 21 individuals, of whom 16 were major industry representatives, 2 were from NGOs, 2 were government representatives, and one was an academic. The networks had very similar profiles, with, for example, 11 of 15 members of the Alcohol Network from major alcohol companies or trade associations, including the Chair [21]. 

**Figure 4:**
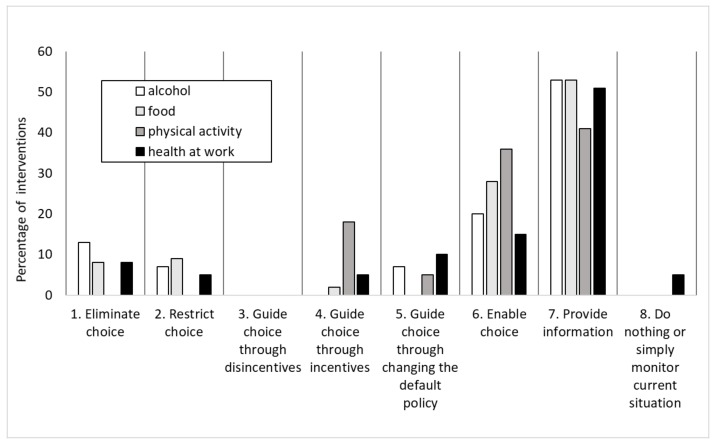
Relative distribution of all RD interventions by type.

Source: authors

Across all networks, individual partners’ preferences in terms of framing and selecting pledges appears to have been to provide information and education to consumers, rather than to implement actions which the evidence suggested would be more likely directly to alter consumer choice and consumption (Figure 4).

As a result, most of the pledges chosen by signatories were found to be likely to have limited impact on health, because they tended not to be drawn from the most effective interventions available, even within a voluntary framework (for example, self-imposed restrictions on marketing, which are known to be effective in relation to food [8] and alcohol consumption [10,11]). This pattern was found within all the networks. For example, in the Food Network, the most effective strategies to improve diet, based on existing evidence, are pricing strategies, restrictions on marketing and reducing sugar intake, but these were not reflected in the RD food pledges. In terms of the “Ladder of Intervention” [30], which ranks the ability of interventions generally to meet public health goals, the alcohol pledges were situated towards the bottom of the ladder, providing information and enabling individual choice [11]. The related evidence review undertaken as part of the evaluation showed such approaches to be of limited effectiveness [10]. Most Physical Activity Network pledges were similarly focused on improving information and individual choice [13]. The Logic Model also illustrated that even if they were achieved, such interventions would represent only small and preliminary steps towards achieving health improvement [23]; information provision needs to be followed by awareness, acceptance, attitude change and behaviour change, and even then there may be no change in individual or population health (including reductions in inequalities).

Another important factor contributing to the lack of public health impact of the RD was that additionality—the ability of the RD to push organisations to make greater changes than would otherwise have been the case—was very limited [4,7,8,11,13]. For example, the pledge analysis found that the large majority of interventions reported by organisations in all four networks appeared to have been already underway without the RD—for example, in the Alcohol Network, only 11% of interventions were likely to have been brought about by the RD; in the Food Network, 26% of pledge-related actions might have been encouraged by the Food Network, and the proportions were much lower in the other networks. The equivalent figures for the Health at Work and Physical Activity Networks were 7%, and 15%, respectively. These findings were corroborated by partner organisations’ accounts in the interviews undertaken as part of the study of RD implementation processes; for example, a key informant from the business sector reported.

*“I believe they wanted people to, kind of, pledge other things, and that kind of thing, but I think you end up with such a long list of pledges, and 99% of them will be what people are doing, anyway”.*

Moreover, a participant in one of the organisational case studies stated that “*To be honest, I think we, the pledges we took were ones we were already delivering”.*

#### 3.2.2. Organisations Involved and Their Rationale for Doing so

**Figure 5:**
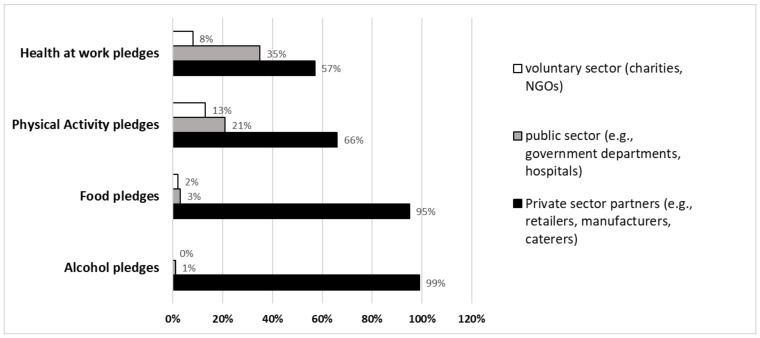
Distribution of RD partners by sector.

Source: authors

Figure 5 shows that the great majority of RD partners, especially in the food and alcohol networks, were recruited from the private sector, and that this is consistent with the RD’s objectives and the logic model and commitments. The key drivers for participation in the RD were captured in the qualitative data and include fulfilling corporate social responsibility commitments, reputational enhancement and, to a lesser extent, staving off the possibility of regulation [9]. The pledge analysis suggests that having joined, the costs to businesses were reduced by making pledges to conduct activities that they were already engaged in, or which they had already planned before they joined the RD [4,7,8,10,11,13]. This supports the finding from the pledge analysis of limited ‘added value’ from the RD, which generally did not appear to lead to “further, faster” progress towards public health goals. Overall, then, the fundamental RD goal was to mobilise the private sector in pursuit of public health improvement, but this was not carried through into pledges that stood any chance of achieving this.

There are obviously potential costs to business, for example, if the implementation of pledges results in customers buying less, or if the development of new products results in reducing sales of other products. This risk appeared to have been reduced by many business partners choosing pledges which were consistent with their business strategy [9], as businesses will not usually voluntarily undertake actions that place them at a competitive disadvantage. This may explain the preference for interventions which involved providing information and education, the likely ineffectiveness of which is well established [4,7,8,10,11,13].

### 3.3. What Were the Feedback Loops and Did They Suppress or Potentiate Effects of the RD on the Outcomes of Interest?

**Figure 6:**
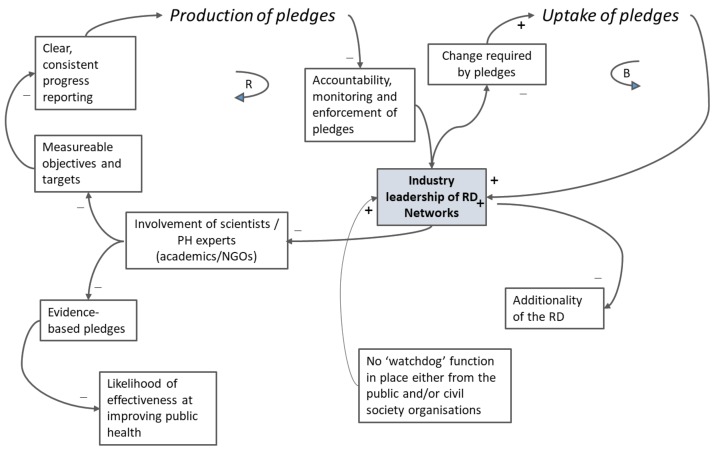
Causal loop diagram of the RD.

**Legend**. A “B“ in the centre of the loop indicates that the loop is balancing; balancing loops oscillate over time. An “R” in the centre of the loop indicates that the loop is reinforcing. Reinforcing loops continually grow or decline over time. A “+” next to a link indicates that one element affects the other element by adding to it or by changing it in the same direction; a “-“ next to a link indicates that one element affects the other element by subtracting from it or changing it in the opposite direction [28]. Source: authors

Figure 6 is a causal loop diagram of the RD system. It demonstrates that there were two competing activities, at the heart of which was a de facto position of leadership by industry of the RD networks (in bold). On the one hand, there was a reinforcing feedback loop (on the left half of the figure) which supported the production of pledges: the design of pledges was led by, or highly influenced by, industry via the networks. This, in turn, resulted in less involvement by public health experts and public health organisations. For example, six public health organisations involved initially in the RD Alcohol Network publicly withdrew their support from the process before the official launch of the partnership. Alcohol Concern, the British Association for the Study of the Liver, the British Liver Trust, the British Medical Association, the Institute of Alcohol Studies, and the Royal College of Physicians expressed concern that the interests of industry were being prioritized at the expense of public health objectives [31]. Other organisations did not get involved because they perceived that it would be resource-intensive (e.g., too much paperwork), or in the case of the non-governmental organisations in public health, because they did not want to participate in agreements which gave industry a large say in making public health policy [9]. Business interviewees also voiced concerns that non-participating businesses could obtain benefit from the RD (such as a competitive advantage) without the costs by not becoming formal partners; there were no sanctions for non-participation. This was referred to as an “uneven playing field” in interviews, and it was also mentioned in the media by some food industry actors who stated that they preferred regulation to voluntary action because it would prevent “free riding” and other strategic behaviour by competitors [9].

The design of the pledges led, or highly influenced, by industry also resulted in few measurable objectives and targets, poor and inconsistent reporting, and poor enforcement of pledge commitments, all of which reinforced the dominance of industry within the RD, and reduced transparency and accountability [4,7,8,11]. Interviewees frequently raised the related consequences of the lack of incentives and sanctions in the RD. The initial scoping literature review had found that in voluntary agreements [12]:

*“Public image plays an important role, both as a benefit and a sanction. …Public image can therefore be used to help encourage compliance. A public announcement of poor performance by a firm within an agreement can also be used as an effective sanction and encourage compliance”.*

This was also found in our analysis of alcohol labelling [6]. Evidence showed that public visibility of voluntary agreements could be an important means to encourage compliance but was lacking in the RD. It is unclear whether this was a direct result of industry dominance over the development of the RD.

On the other hand, there is a balancing feedback loop (on the right half of Figure 6) which supported the uptake of pledges. Yet, we found that this meant weaker, less clearly defined pledges [4,7,8,10,11,13], which did not explicitly require signatories to make major changes, as noted above. This supported the uptake of pledges, mostly by industry members (Figure 5), and reinforced the leadership of industry in the RD.

We considered also the likely interaction between pledges, and whether their collective public health effect might be more positive than the sum of the individual pledges would suggest. However, as the choice of pledges showed a strong tendency towards interventions whose ineffectiveness is well known, this seems highly unlikely. None of the qualitative or quantitative evidence supported a synergistic interpretation of the totality of the pledges. This does not, of course, rule out the possibility that if more effective individual pledges had been selected, they might, in principle, have contributed to more effective multi-component programmes (a point we made in our evaluation of the Health at Work pledges [32].

### 3.4. How Resilient Was the System to Change and What was Its Ability to “Absorb” Externally Directed Change?

If the system in which the public health intervention is operating is so resilient that it can *absorb* that intervention and “spring back into shape”, it renders the intervention ineffective. Overall, the system within which the RD was operating was resistant to change because those leading the design of the RD—i.e., businesses—did not, by definition, have public health as a primary interest; the proposed actions themselves were therefore ineffective; and no external watchdog function was in place to hold the government and RD leads to account [4,7,8,9,11]. Therefore, the wider alcohol, food, and other systems (and subsystems) which the RD was supposedly targeting were effectively unchanged by its intervention. One could argue that these systems’ ability to self-organise to their advantage by creating or influencing the structures and processes of the RD, as demonstrated above, made them resilient to any interventions which do not meet their primary goals. From the participating businesses’ perspective, this may mean that the RD was a success, because in participating they managed to persuade government that they were part of the solution, shaped the intervention (in some cases delaying or postponing regulatory action which might have had an impact on profitability). From a public health perspective, however, the RD was not a success—as there was little evidence from any network that the RD had achieved much for public health. Its existence may have made public health improvements in the relevant areas harder, at least in the short to medium term. 

## 4. Discussion

Returning to our initial definition of a system, we suggest that the RD system is made up of a set of *elements* including actors involved in designing the RD, the RD partners, the evidence on the effectiveness of specific interventions to improve public health, and the desire on the part of both Government and business for businesses to be seen as “part of the solution”. These elements were *interconnected* through mechanisms (feedback loops) in such a way that they produced their own pattern of behaviour over time; in this case, the behaviour was such that the business RD partners had a dominant say in the design and execution of the RD, resulting in a systemic resistance to meaningful change in public health terms. Finally, the *function or purpose* of the RD was to support a paradigm of market-driven behaviours, where it is fundamentally down to the individual to change. This was clearly reflected in the nature of the pledges which emphasised better informing the consumer. 

### 4.1. The RD in the Context of the Wider Literature on Public-Private Partnerships

The findings are consistent with the wider literature on self-regulation and voluntary agreements in public health [33,34,35,36,37]. The RD approach fits within a spectrum of such agreements [38], towards the “industry self-regulation” end of the spectrum.

This literature demonstrates that industry self-regulation and voluntary agreements or partnerships between government and industry have repeatedly been shown to be ineffective public health policy mechanisms [33,34,35]. This has also been shown in previous evaluations of specific food and alcohol-related self-regulation and voluntary agreements [36,37]. These find that the codes or agreements themselves are usually based on commitments which are vague, and focused on education and information for consumers, while being hampered from a public health policy perspective by limited monitoring and reporting [39]. Finally, self-regulation and voluntary approaches are usually based on voluntary participation (so do not involve all relevant industry players) [34]. As a result, any claims about the effectiveness and acceptability of voluntary agreements need to take account of this, as well as the likely variation in compliance [39]. It is noteworthy that U.K. retailers and manufacturers have been asking for stronger measures from government to achieve “a level playing field” and avoid a “piecemeal response from business” [9]. It has also been suggested more fundamentally that health-related public-private partnerships risk reorienting public health policy away from its core focus on the public’s health, as a result of policy or institutional capture by industry partners [40].

The findings of the RD evaluation also contribute new evidence relevant to the development, implementation and evaluation of more effective voluntary agreements in future, if governments continue to wish to pursue them. They show that there is a high risk that such agreements will encourage firms to agree to actions that they were doing already, and especially actions which do not risk negatively affecting sales, or consumer perceptions and behaviour. Thus explicit steps need to be taken to ensure and monitor additionality. They also suggest that such agreements require robust independent monitoring, and careful setting of specific pledges with quantifiable, unambiguous, time-bound outcome targets. Both our scoping review and subsequent evaluation further underline that lack of independence in monitoring is highly problematic. Self-monitoring without independent verification of claims undermines trust in the agreements and undermines the overall plausibility of any claims about effectiveness. Lack of independent verification also significantly raises the risk of reporting bias (overstatement by businesses of the effectiveness of their actions).

Buse et al. [2] have suggested that governance of the commercial determinants of health in order to reduce the risk of non-communicable disease is currently weak, and frequently brings public health into conflict with the interests of profit-driven food, beverage, alcohol and tobacco industries. They have suggested that where self-regulation is used, and it is still frequently the only or preferred option of governments in many countries, a set of policy design features is needed for such approaches to have any chance of having public health impact, which include:

(1) evidence-informed and rights-based targets that are in the interest of public health, and that are developed in a transparent manner and SMART in design (i.e., specific, measurable, attributable, realistic and time-bound);
(2) independent, rigorous monitoring of compliance, progress and public health impact;
(3) transparent reporting with clear consequences and independent measures of accountability to the government and to the public;
(4) sufficient scope for impact, through the participation of leading corporate players and applied as widely as possible to cover the largest possible proportion of those exposed to risk of ill-health and the market.
(5) range of contributors: “…self-regulatory regimes should have inputs from governments, scientists and civil society, particularly in target and standard-setting and ensuring accountability” [2].

Conditions (1), (2), and (3) were not met in the case of the RD. While (4) was largely met, our analysis shows that it is easily circumvented. The alcohol, food, physical activity and workplace health sub-systems which the RD was targeting were on the whole unchanged by the intervention. Interest groups and individuals from these wider systems were part of the RD intervention, chose how it was to be implemented, shaped its success criteria, and produced their own (largely unmonitored) evidence of how it was working. These wider economic systems, in the end, easily absorbed and largely nullified any commercial threat which the RD might have posed. 

The main limitations of our original evaluation, on which this paper draws, related to the variable reporting by participating organisations, and the lack of quantitative monitoring measures across all pledges [11]. Also there may have been studies we did not locate despite our systematic reviews of the literature [8,10]. Finally a limitation was the number of interviews conducted [9]; however this was offset by being able to capture a wide range of partner organisations and stakeholders involved in the design and implementation of the RD, therefore getting a nuanced set of perspectives on the RD [9]. The strength of the current paper is a worked example of how a complex public health policy such as the RD can be evaluated through a systems lens, and health can be conceptualized as the outcome of the interconnection of different elements (such as the interests of specific actors). It is hoped that this approach helps to inform the design of independent public health policies with population health as the main interest at stake.

## 5. Conclusions

This paper uses a systems approach to integrate for the first time the evidence from the previously distinct but linked strands of the RD evaluation. Doing this demonstrates even more clearly than otherwise how and why the RD did not meet its stated objectives to improve public health in England. Both the production and uptake of pledges by partners were largely driven by the interests of partners themselves, enabling the wider business systems within which the RD was operating to remain resistant to change. 

## Figures and Tables

**Table 1:** Components of the Public Health Responsibility Deal (RD) evaluation, and aspects of the system which they shed light on.

RD Evaluation Components	System Attributes which these Data Illuminate (Adapted from [20])
Logic model built on initial description of how RD would work [23], and scoping review [12]	Causal pathways within the RD systems (food, alcohol, physical activity, health at work)
Participant interviews [9] Analysis of organisational case studies including documents and interviews Media analysis [24]	Structures and processes in place Interests at play Feedback loops, and barriers to change
Qualitative systems dynamic modelling [15,25,26] using Causal Loop Diagrams (as an analytic tool) [27] built on data from pledge analyses, progress report analyses, qualitative data from interviews and organisational case studies (created as part of the current paper)	Drivers, interests, ways of working
Analysis of RD pledges [4,5,6,7,8,9,10,11,13,24]	
Analyses of evidence base [4,5,6,7,8,9,10,11,13,24]	Probability of system changing in response to specific pledges
Analyses of specific pledges [4,5,6,7,8,9,10,11,13,24]	Identifying whether change happened in a particular part of the system

## Abbreviations

CLD	causal loop diagram
PPP	public–private partnership
RD	the Public Health Responsibility Deal
SMART	specific, measurable, attributable, realistic, and time-bound
